# The Biosynthesis of Heterophyllin B in *Pseudostellaria heterophylla* From *prePhHB*-Encoded Precursor

**DOI:** 10.3389/fpls.2019.01259

**Published:** 2019-10-17

**Authors:** Wei Zheng, Tao Zhou, Jun Li, Weike Jiang, Jinqiang Zhang, Chenghong Xiao, Dequn Wei, Changgui Yang, Rong Xu, Anhui Gong, Chen Zhang, Yan Bi

**Affiliations:** ^1^Experiment Center, Guizhou University of Traditional Chinese Medicine, Guiyang, China; ^2^Graduate School, Tianjin University of Traditional Chinese Medicine, Tianjin, China; ^3^School of Pharmacy, Guizhou University of Traditional Chinese Medicine, Guiyang, China

**Keywords:** cyclic peptide, heterophyllin B, gene, *prePhHB*, *Pseudostellaria heterophylla*

## Abstract

Plant cyclic peptides (CPs) are a large group of small molecule metabolites found in a wide variety of plants, including traditional Chinese medicinal plants. However, the majority of plant CPs have not been studied for their biosynthetic mechanisms, including heterophyllin B (HB), which is one of the characteristic chemical components of *Pseudostellaria heterophylla*. Here, we screened the precursor gene (*prePhHB*) of HB in *P. heterophylla* and functionally identified its correctness *in vivo* and *in vitro*. First, we developed a new method to screen the precursors of HB from 16 candidate linear peptides. According to transcriptome sequencing data, we cloned the genes that encoded the HB precursor peptides and confirmed that the *prePhHB*-encoded precursor peptide could enzymatically synthesize HB. Next, we generated the transgenic tobacco that expressed *prePhHB*, and the results showed that HB was detected in transgenic tobacco. Moreover, we revealed that *prePhHB* gene expression is positively correlated with HB accumulation in *P. heterophylla*. Mutations in the *prePhHB* gene may influence the accumulation of HB in *P. heterophylla*. These results suggest that HB is ribosomally synthesized and posttranslationally modified peptide (RiPP) derived from the precursor gene *prePhHB*-encoded precursor peptide, and the core peptide sequence of HB is IFGGLPPP in *P. heterophylla*. This study developed a new idea for the rapid identification of *Caryophyllaceae*-type CP precursor peptides *via* RNA-sequencing data mining.

## Introduction

Bioactive peptides have a wide range of physiological functions, including regulating signal transmission, promoting enzyme synthesis, and resisting virus invasion (Jhamandas and Goncharuk, 2013; Noh et al., 2015). Cyclic peptides (CPs) are a special type of active peptides that have a circular molecule structure (Daly et al., 2006; Otzen, 2017). CPs are distributed in a very wide range of taxa, including plants, bacteria, fungi, and animals (Tan and Zhou, 2006; Cascales and Craik, 2010; Burman et al., 2014). They have a diverse range of biological activities, including anti-inflammation, antibacterial, and anti-HIV activities (Henriques et al., 2011; Raju et al., 2014; Noh et al., 2015). In addition, CP compounds can form tubular structures and act as membrane ion transferring carriers (Rodriguez-Vazquez et al., 2014). Plant CPs are a large group of small molecule metabolites, typically with 2–37 protein and non-protein amino acids (mainly L-amino acids), which are formed mainly by the peptidic bonds and isolated from the stem barks, leaves, seeds, and roots of a wide variety of higher plants (Condie et al., 2011; Xu et al., 2012). So far, more than 450 plant CPs have been discovered from 26 families of higher plants; they are most commonly found in the *Caryophyllaceae*, *Rhamnaceae*, and *Violaceae* families (Tan and Zhou, 2006; Burman et al., 2010; Pandey et al., 2012).

Several traditional Chinese medicinal plants contain abundant plant CPs, including *Pseudostellaria heterophylla* (Miq.) Pax (Morita et al., 1995; Han et al., 2008). *P. heterophylla* is an herbaceous perennial, a *Caryophyllaceae* species known as Taizishen (Hua et al., 2016a). Its dried tuberous root is named Pseudostellariae radix and is a traditional Chinese medicine with high pharmacodynamic value. It can be used to treat spleen deficiency, anorexia, weakness after illness, and spontaneous perspiration symptoms because of its various active components, including saponins, polysaccharides, and cyclopeptides (Pang et al., 2011; Hu et al., 2013; Wang et al., 2013). Heterophyllin B (HB) is one of the characteristic chemical components of *P. heterophylla* and is used as the quality control index for evaluating Pseudostellariae radix in the Chinese pharmacopoeia (2010 edition, Volume I) (Zhao et al., 2015). HB is a cyclic octapeptide with a single ring formed with peptide bonds and eight L-amino acids, which belongs to the *Caryophyllaceae*-like CPs and is abundant in the tuberous roots of *P. heterophylla* (Zhao et al., 2015). Studies have shown that HB is effectively suppressed the adhesion and invasion of the human esophageal carcinoma cells and ameliorates lipopolysaccharide-induced inflammation and oxidative stress in macrophages by mediating the PI3K/AKT/β-catenin pathways (Tantai et al., 2016; Yang et al., 2018). However, the mechanism for the biosynthesis of HB is still unclear.

Fortunately, recent studies have found that CPs are generated through two biosynthetic routes, which involve either non-ribosomal peptide synthases or ribosome-dependent production of precursor peptides. In one route, ribosomes are participated in the initial ordering of mRNA-encoded amino acids to form a linear peptide precursor (Schmidt et al., 2005; Gillon et al., 2008; Arnison et al., 2013; Bionda and Fasan, 2017). In another route, the amino acids are ordered without direct ribosome involvement, *via* non-ribosomal peptide synthetases (Finking and Marahiel, 2004). Accumulated evidence suggests that ribosome-dependent biosynthesis of CPs were widespread in animals and fungi (Walton et al., 2010; Luo et al., 2012; Cai et al., 2016). Moreover, ribosome-dependent biosynthesis of CPs also have been reported in plant. Segetalins, belonging to *Caryophyllaceae*-like CPs, have been demonstrated to be formed by ribosome-dependent linear peptides (Condie et al., 2011). It has also been reported that a small amount of cyclized product (HB) was produced when linear peptide NH2-Gly^1^-Gly^2^-Leu-Pro-Pro-Pro-Ile-Phe-COOH of HB was incubated with crude enzyme extracted from *P. heterophylla* (Jia et al., 2006; Xu et al., 2012). In addition, plant genomes do not encode nonribosomal peptide synthetases (NRPSs) (Kersten and Weng, 2018). Therefore, we hypothesized that HB is biosynthesized from ribosome-derived linear precursors.

In the present study, we developed a new approach to screen the precursor genes (*prePhHB*) of HB in *P. heterophylla*. It was demonstrated *in vivo* and *in vitro* that linear peptides encoded by *prePhHB* could eventually synthesize HB. Moreover, it is probable that mutations in the *prePhHB* gene affect HB synthesis in *P. heterophylla*.

## Materials and Methods

### Plant Materials

Sample collection was performed for *P. heterophylla* cultivated in five areas, including Jiangsu Province, Fujian Province, Anhui Province, Guizhou Province, and Shandong Province, China. Among them, three samples were collected from per cultivated area. These samples were used to compare the content of HB. All sample information is shown in **Table S1**.

Regarding transplantation, *P. heterophylla* from the above four areas (the specific location is shown in **Table S2**) were planted in Guizhou (107°55′31″N, 27°8′24″E) for 1 year. After that, these plants of *P. heterophylla* were used for gene sequence analysis and HB analysis.

Wild-type tobacco seeds were surface-sterilized, planted on Murashige and Skoog (MS) medium, and solidified with 3% (w/v) sucrose and 0.7% (w/v) agar. Six-week-old tobacco plants, which were grown in an intelligent artificial climate box under long-day conditions (16 h light/8 h dark) at 25°C, were used for identification of the *prePhHB* gene.

### Character Analysis

Sampling was performed according to the quartering method. The weight of single fresh tuberous root of 138 P. *heterophylla* samples was determined through an electronic balance. Twenty tuberous roots were measured for each sample. The weight accuracy is 0.01 g.

### High-Performance Liquid Chromatography (HPLC) Analysis

All samples were dried at 60°C up to dryness *via* the oven-drying method and crushed to a mediate powder. Next, 0.5 g of the mediate powder in 25 ml of ethanol was ultrasonicated for 45 min and filtered. Next, 20 ml of the filtrate was evaporated to dryness; the residue was dissolved in 5 ml of ethanol, which was passed through 0.45-µm syringe filters to obtain the test solution and analyzed *via* HPLC. HB standard (purity ≥ 95%) was obtained from Laboratory of Phytochemistry, Kunming Institute of Botany, Chinese Academy of Sciences (Kunming, China).

HPLC analysis of HB was performed on a Shimadzu LC-20AD Prominence HPLC System (Shimadzu, Japan) that consisted of a LC-20AD Pump, a SIL-20A Autosampler, an SPD-M20A DAD, and a CTO-20AC column heater, using a Pntulips™ QS-C18 column (4.6 × 250 mm, 5-μm particle size). The mobile phase was acetonitrile: H_2_O (31: 69, v/v). The flow rate was kept at 1.0 ml/min, and the eluate was injected onto a DAD detector. The injection volume of the test solution was 20 µl, and the injection volume of HB standard was 10 µl. The column temperature was maintained at 30°C, and the wavelength of the detector was set at 192 nm. HB was identified *via* its retention time and spectral data compared with those of HB standard, and the content of HB in the test solution was calculated *via* the one-point external standard method.

### Total RNA Extraction and cDNA Synthesis

Total RNA was extracted from each sample using a RNAiso Plus Kit (TaKaRa, Japan) according to the manufacturer’s instructions. RNA integrity was determined using 1.5% agarose gel; RNA concentration and purity were assessed on a Nanodrop 2000 spectrophotometer (Thermo Fisher Scientific, USA). Next, 800 ng of total RNA from each sample was reverse-transcribed into single-stranded cDNA using a M-MLV Reverse Transcriptase Kit (TaKaRa, Japan) and oligo (dT)_15_ primer, following the manufacturer’s instructions. The first-strand cDNA was diluted to a final concentration of 80 ng·μl^−1^ and used as templates for PCR and real-time quantitative polymerase chain reaction (RT-qPCR).

### DNA Extraction

DNA was extracted from the kanamycin-resistant tobacco plants using the modified CTAB method (Arseneau et al., 2017). And the presence of the *prePhHB* gene was demonstrated *via* PCR analysis.

### Gene Screening

Considering that the cyclization pattern of Caryophyllaceae-like CPs is head-to-tail, their nomenclature is written as a one-line text formula, including the prefix “cyclo,” a hyphen and a sequence of amino acids in a particular order (Spengler et al., 2005; Arnison et al., 2013). In the case of HB, being a cyclic octapeptide, 16 possible symbols after ring-opening can be used to write its single line formula (**Table 1**). They were screened for exact matches from six-frame translations of the *P. heterophylla* RNA-sequencing (RNA-seq) database (Li et al., 2016). Finally, similarity analysis was performed between the predicted amino acid sequences of the retrieved cDNAs and the amino acid sequences of known CP precursor (Condie et al., 2011).

**Table 1:** Sixteen possible amino acid sequences after the ring-opening of the cyclic octapeptide HB; these were screened for exact matches from six-frame translations of the *P. heterophylla* RNA-seq database.

No.	Amino acid sequences	Number of unigene	Unigene number	No.	Amino acid sequences	Number of unigene	Unigene number
1	IFGGLPPP	1	c57752_g2	9	PPPLGGFI	0	0
2	FGGLPPPI	0	0	10	IPPPLGGF	0	0
3	GGLPPPIF	0	0	11	FIPPPLGG	0	0
4	GLPPPIFG	0	0	12	GFIPPPLG	0	0
5	LPPPIFGG	0	0	13	GGFIPPPL	0	0
6	PPPIFGGL	0	0	14	LGGFIPPP	0	0
7	PPIFGGLP	0	0	15	PLGGFIPP	0	0
8	PIFGGLPP	0	0	16	PPLGGFIP	0	0

### Isolation of the Full-Length cDNA of *prePhHB*

A pair of primers, *prePhHB*-F/*prePhHB*-R (**Table 2**), were designed to amplify the unigene c57752_g2 from *P. heterophylla* (“GZSB”) for sequence verification. The amplified PCR products were directly cloned into pMD19-T vector (TaKaRa, Japan) and then transformed into *Escherichia coli* DH5α competent cells (Tiangen, China) according to the manufacturer’s instructions. Putative recombinant clones were screened *via* PCR using M13 primers (**Table 2**), and the positive clones were further confirmed *via* Sanger sequencing at Nanjing GenScript Biotech Co., Ltd. (Nanjing, China).

**Table 2:** Primers used to isolate HB precursor genes from *P. Heterophylla, M13-R and M13-F*.

Primer name	Sequence (5′→3′)
T15	TTTTTTTTTTTTTTT
*prePhHB*-F	ATGTCTACTATTTCAGCC
*prePhHB*-R	CTTACACCATGAGGAAA
*prePhHB*-5	AGGAGGAGGAAGACCCCCAAAAAT
*prePhHB*-3	ATTTTTGGGGGTCTTCCTCCTCCT
Long primer	CTAATACGACTCACTATAGGGCAAGCAGTGGTATCAACGCAGAGT
CDS-*prePhHB*-F	AGTATTTTTGGGGGTCTTCCTCCTC
CDS-*prePhHB*-R	TACACAACACCATAAAAGCCCAACG
*prePhHB*-S	CGCGGATCCATGTCTACTATTTCAGCCATCCAC
*prePhHB*-A	CCGGAATTCTTACACCATGAGGGAAATATCATC
*prePhHB*-qF	AGCCGAGTATTTTTGGGGGTCTTCC
*prePhHB*-qR	CATAAAGGCCCAACGGTGACGGAC
*PhACT2*-F	CTCCATACCGATAAATGAAGGC
*PhACT2*-R	CACTGTTCCAATCTATGAGGGTTA
QX-F	ACGACTCAATGACAAGAAGAA
QX-R	CCGGCGGTAAGGATCTGA
Actin-qF	CAGCAAAGACCAGCTCATCC
Actin-qR	AGCAGCTTCCATTCCGATCA
M13-R	CAGGAAACAGCTATGAC
M13-F	GTAAAACGACGGCCAG

After DNA sequencing analysis, two RACE specific primers, *prePhHB*-5 and *prePhHB*-3 (**Table 2**), were designed based on the 108-bp singlet to clone the 5’- and 3’- ends of the *prePhHB* cDNA *via* rapid amplification of cDNA ends (RACE) using the SMARTer® RACE 5’/3’ Kit (TaKaRa, Japan), according to the manufacturer’s instructions. Other experimental methods are shown above.

### Isolation of the Coding Sequence of *prePhHB*

To determine whether the newly identified *prePhHB* exhibits sequence differences in the differently cultivated *P. heterophylla*, one pair of specific primers, CDS-*prePhHB*-F/CDS-*prePhHB*-R (**Table 2**), was designed for specific combination with the *prePhHB* sequence which the core peptide of HB. The cloning procedure used was the same as described above. Lastly, positive clones (named *prePhHB*-JSJR, *prePhHB*-AHXZ, *prePhHB*-GZSB, and *prePhHB*-FJZR, respectively) were picked out and sent to GenScript (Nanjing, China) and Sangon Biotech (Shanghai, China) for sequencing.

### Phylogenetic Analysis

Multiple alignments of amino acid sequences were performed using the ClustalX (version 2.1) software, and then, the neighbor-joining phylogenetic tree was constructed *via* using MEGA 6.0 software. Branch points were assessed by bootstrap analysis with 1,000 replications.

### Enzymic Synthesis of HB *In Vitro*

The linear precursor of HB encoded by *prePhHB* gene was synthesized using the FlexPeptide™ technology by GenScript (Nanjing, China). Five grams of phloem of the tuberous roots of “GZSB” were manually homogenized with a ceramic pestle in a 100-mm ceramic mortar in 4 × 5 ml 20 mM Tris-HCl (pH 8.0) on ice and transferred to a 50-ml sterile microcentrifuge tube. Next, 2 ml 20 mM Tris-HCl (pH 8.0) solution was used to clean the mortar and was transferred to the 50-ml sterile centrifuge tube. Shaking and extraction were performed at 100 rpm for 30 min, followed by centrifugation at 8,000 rpm for 30 min. The supernatant was centrifuged using 100-kDa ultrafiltration centrifuge tube and then using 30-kDa ultrafiltration centrifugal tube. The concentrate collected was then added with an appropriate amount of 1 M Tris-HCl (pH 8.5), shaken, and centrifuged. This crude extract supernatant (i.e., crude enzymes) was used for *in vitro* functional assays of linear precursor of HB. The crude enzymes were identified *via* SDS-PAGE and measured using a BCA Protein Assay Kit (CWBIO, China).

An *in vitro* functional assay for linear precursor of HB was performed in a 2-ml reaction mixture that contained 20 mM Tris-HCl (pH 8.5), 100 mM NaCl, 5 mM DTT, 0.2 mg BSA, and 25 μg·ml^−1^ linear precursor of HB. The reaction was initiated by the addition of 1 mg crude enzyme. Five different reaction times and the temperature range of 25 to 37°C were screened to determine the optimum time and temperature condition for the reaction. At the indicated times, 2 ml of reaction mixture was removed and stopped *via* placing reactions in dry ice. The samples were then filtered using 0.22-μm filters, and a 2-ml filtered sample was analyzed *via* HPLC (see above).

### Construction of the *prePhHB* Expression Vector and Genetic Transformation

The full-length coding sequence of *prePhHB* with specific restriction enzyme sites (**Table 2**) was cloned into the pLGNL vector at the BamH I and EcoR I sites to obtain the transformation vector pLGNL-*prePhHB*. Therefore, a fragment of *prePhHB* was driven by the cauliflower mosaic virus 35S promoter. All constructs were verified *via* DNA sequencing.

After sequence verification, the resultant plasmid (pLGNL-*prePhHB)* was transformed into the *Agrobacterium tumefaciens* strain LBA4404 competent cells (Weidi, China) using the freeze-thaw method. And then transferred into tobacco plants *via* *Agrobacterium*-mediated transformation following the protocol described by Pathi (Pathi et al., 2013). Next, transgenic lines were selected on a selective medium containing 50 mg/L kanamycin and 100 mg/L cefotaxime sodium salt and transferred to soil and grown until seed harvest.

### Ultra-High-Performance Liquid Chromatography–Mass Spectrometry (UHPLC-MS/MS) Analysis

The roots, stems, and leaves of transgenic lines (L1, L22, and L25) along with WT (wild-type tobacco, non-transgenic) plants were dried to dryness at 60°C *via* the oven-drying method and then crushed to mediate powder. Next, 2 g of the mediate powder in 50 ml of ethanol was ultrasonicated for 45 min and filtered. Then, 25 ml of the filtrate was evaporated to dryness. The residue was dissolved in 5 ml of ethanol and filtered through a 0.22-µm syringe filter into a sample vial for subsequent analysis.

Thermo Scientific UHPLC Accela 1250 System was connected to a Thermo Scientific TSQ Quantum Access MAX Mass Spectrometer (San Jose, CA, USA) to perform the analysis. The chromatographic separation was performed using a Thermo Scientific UHPLC Accela 1250 System consisting of an Accela 1250 PDA Detector, an Accela HTC PAL Autosampler, and an Accela 1250 pump. The extract was applied to a Hypersil GOLD column (50 mm × 2.1 mm, 1.9 μm) maintained at 45°C. The mobile phase was acetonitrile: 0.1% formic acid/water (30:70, v/v). The injection volume was 5 µl, and the flow rate was kept at 0.2 ml/min.

Mass spectrometric detection was carried out on a Thermo Scientific TSQ Quantum Access MAX Mass Spectrometer, which was equipped with an electrospray ionization (ESI) interface operating in positive ion mode. The parameters were set as follows: the pressure of nebulizing gas (N_2_) was set at 30 and 5 arbitrary units (Arb), respectively; capillary temperature, 350°C; nebulizing temperature, 500°C; spray voltage, 2500 V; and scanning frequency, 0.1 s. The selected reaction monitoring (SRM) transitions were monitored at m/z 69.52 → 183.10. Data acquisition was performed on the LCQUAN™ quantitation software.

### RT-qPCR

RT-qPCR primers are listed in **Table 2**. RT-qPCR was performed using a SYBR® *Premix Ex Taq*™ II (Tli RNaseH Plus) (TaKaRa, Japan) with an Applied Biosystems 7500 Real-Time PCR System (Applied Biosystems, USA). All reactions were performed in three biological replicates per sample with three technical replicates each. Relative expression of *prePhHB* in each sample was calculated using the 2^−ΔCT^ method (Ballester et al., 2013). The *PhACT2* (GenBank accession number KT363848) and *ACTIN* (GenBank accession number AY179605) genes were used as housekeeping genes, respectively.

### Statistical Analysis

All results are expressed as the means ± standard errors of mean (SEM). Statistical analysis and graphs were performed using GraphPad Prism (version 7.0). Potential differences between the mean values were evaluated using a one-way analysis of variance (ANOVA) followed by the least significant difference (LSD) test for *post hoc* comparisons when equal variances were assumed. P < 0.05 was considered as statistically significant difference.

## Results

### The Content of HB in *P. Heterophylla* Is Different in Five Planting Regions

**Figure 1:**
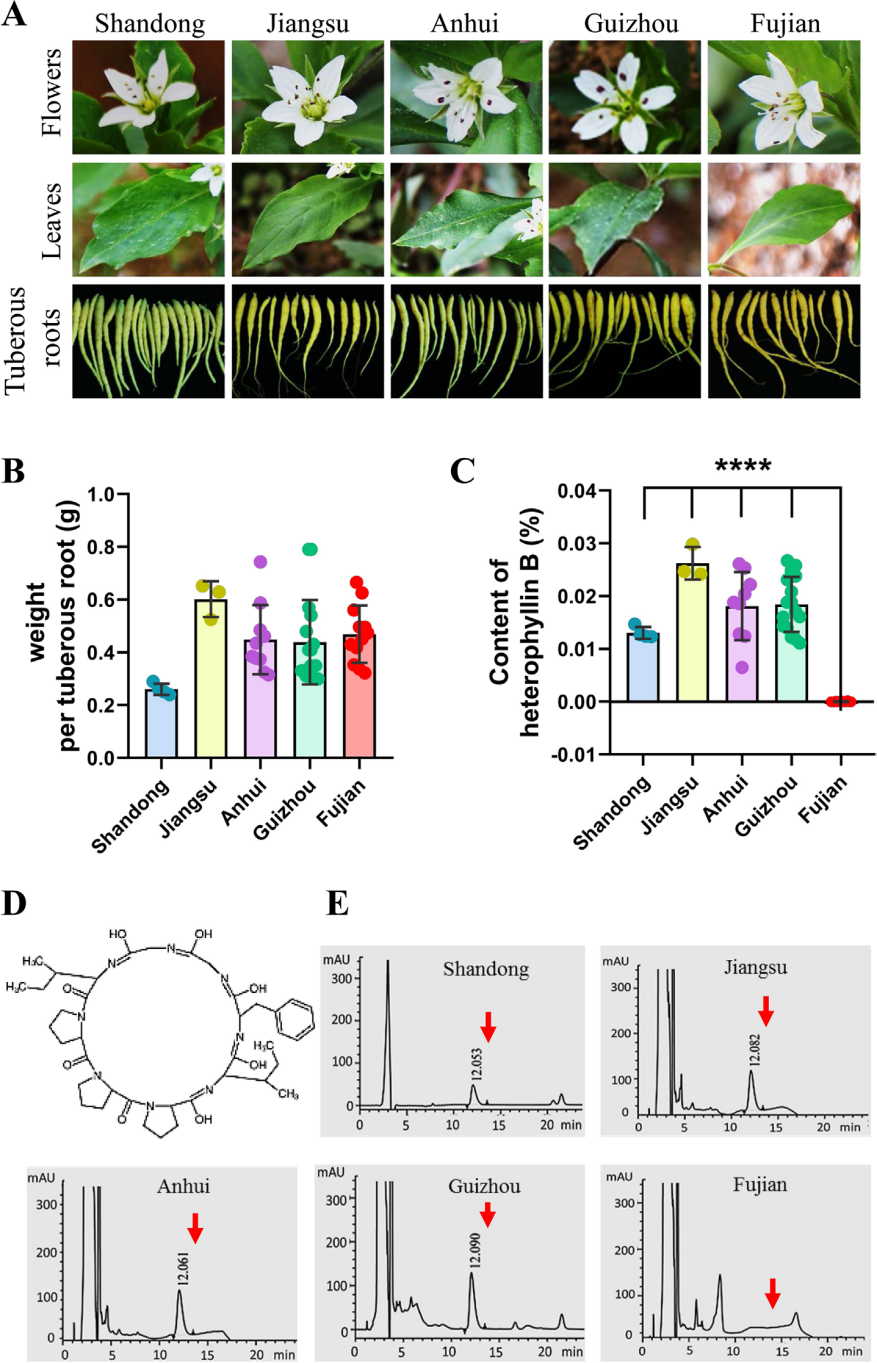
There is a difference in the content of HB of *P. heterophylla* with respect to the areas of major cultivation in China. **(A)** Morphological pictures of the flowers, leaves, and tuberous roots of cultivated *P. heterophylla* in five areas, including Jiangsu Province, Fujian Province, Anhui Province, Guizhou Province, and Shandong Province, China. **(B)** Analysis of fresh weight per tuberous roots in five provinces of China. Small circles of different colors were shown as the means of 3 biological replicates each containing 20 tuberous roots, and bars represent means ± SEM of 4 independent regions for “Shandong,” 3 independent regions for “Jiangsu”, 8 independent regions for “Anhui”, 16 independent regions for “Guizhou”, and 13 independent regions for “Fujian”. **(C)** The content of HB in the tuberous root of cultivated *P. heterophylla* in five provinces. Small circles of different colors also represent means of three biological replicates, and bars represent means ± SEM of n independent regions (n = 4 for “Shandong”, n = 3 for “Jiangsu”, n = 8 for “Anhui”, n = 16 for “Guizhou”, n = 13 for “Fujian”). Data between provinces (Shandong Province *vs*. Fujian Province, Jiangsu Province *vs*. Fujian Province, Anhui Province *vs*. Fujian Province, Guizhou Province *vs*. Fujian Province) were analyzed by one-way ANOVA, and **** denotes statistical significance at p < 0.0001. **(D)** Structure of HB (a cyclic octapeptide). **(E)** HPLC analysis of HB in tuberous roots of cultivated *P. heterophylla* from five provinces. The structure of HB, corresponding to the peaks, is marked by the red arrow.

At present, in the traditional Chinese medicine market, the cultivated *P. heterophylla* are mainly produced from five provinces of China, including Shandong, Jiangsu, Anhui, Guizhou, and Fujian. We collected the samples of *P. heterophylla* from these provinces and measured the fresh weight of single tuberous root. No significant differences were found when we compared the characters of flowers, leaves, and tuberous roots of *P. heterophylla* (**Figure 1A**). However, the results showed that the fresh weight of tuberous roots also had no statistically significant difference in these *P. heterophylla* samples (**Figure 1B**).

HB is one of the characteristic chemical components of *P. heterophylla*, which is a cyclic octapeptide with a single ring formed with peptide bonds and eight L-amino acids (**Figure 1D**). We further detected the HB content in tuberous roots of cultivated *P. heterophylla* from different areas using HPLC (**Figure 1E**). Surprisingly, even though under the same detection conditions, HB was not detected in the cultivated *P. heterophylla* from Fujian province (**Figures 1C, E**). This result indicates that the accumulation of HB in *P. heterophylla* has a distinct regionality. We also speculated that this regionality may be related to the ecological environment and the germplasm genetic factors of *P. heterophylla*.

To eliminate the influence of environmental factors on the accumulation of HB in *P. heterophylla*, we introduced *P. heterophylla* seedling from these four provinces (Fujian, Jiangsu, Anhui, and Guizhou) into the same environment (**Table S2**). We also detected the content of HB in tuberous roots of *P. heterophylla*. The results were similar to those found above; it is suggested that HB content differences may be mainly related to the germplasm genetic factors.

### Screening of the *prePhHB* Gene-Encoding Linear Precursor of HB From *P. Heterophylla*

**Figure 2:**
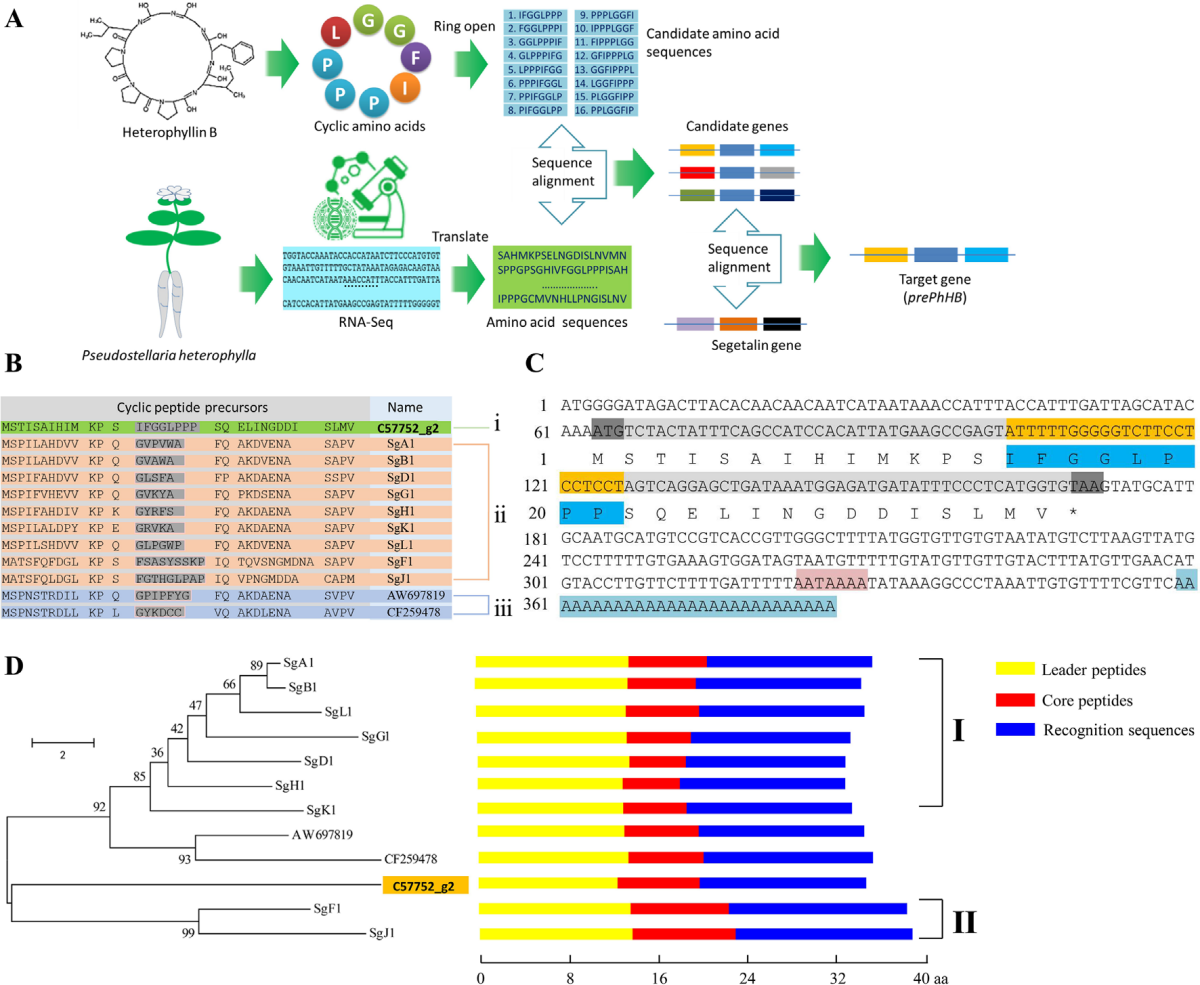
Screening and identification of the preheterophyllin B gene from the *P. heterophylla* RNA-seq database. **(A)** Schematic diagram of the preheterophyllin B gene acquisition. **(B)** Manual alignment of the predicted amino acid sequences of cDNAs encoding putative CPs of (i) *P. heterophylla*, (ii) *Saponaria vaccaria*, and (iii) *Dianthus caryophyllus*. Core peptide motifs are shown in gray. **(C)** Nucleotide and deduced amino acid sequences of the *prePhHB* gene. The start codon (ATG) and the stop codon (TAA) are shaded with gray. The nucleotide sequences (yellow) and deduced amino acid sequences (dark blue) of the core peptide motif are shown. The AATAAAA frame is shaded in pink. Blue indicates the poly (A) tail. **(D)** Phylogenetic analysis and motif analysis of the amino acid sequences that encode the *Caryophyllaceae*-like CP precursors. Class I are precursors of segetalin A, segetalin B, segetalin D, segetalin G, segetalin H, segetalin K, and segetalin L of *S. vaccaria*. Class II is precursors of segetalin F and segetalin J of *S. vaccaria*. Schematic diagram for the motifs is indicated on the right of each precursors. (Yellow: leader peptide motifs, red: core peptide motifs, blue: recognition sequence motifs).

To obtain the gene encoding the linear precursor of HB, we opened the ring of HB to 16 possible linear peptides. These linear peptides were matched to amino acid sequences which translated from the *P. heterophylla* RNA-seq database *via* six-frame translations (**Figure 2A**). The results showed that the amino acid sequence no. 1 was matched to one unigene (i.e., c57752_g2) (**Table 1**). To further identify the unigene c57752_g2, we used the putative amino acid sequence of the unigene (c57752_g2) to perform a homologous comparison with the *Caryophyllaceae*-like CP precursors of *Saponaria vaccaria* and *Dianthus caryophyllus* (accession numbers AW697819 and CF259478). The results from multiple amino acid sequence alignment and amino acid composition analysis showed that there is a high similarity between the 12 amino acid sequences (**Figure 2B**). These results suggest that the unigene c57752_g2 may be a precursor gene encoding the linear precursor of HB, which is named *perPhHB*.

Based on the known fragment of *perPhHB*, specific primers for RT-PCR, 5′-RACE, and 3′-RACE were designed to isolate the full-length *perPhHB* cDNA sequence from *P. heterophylla*. The full-length cDNA (386 bp) contained a 108-bp coding sequence, which encodes a putative precursor of HB with 35 amino acids, a 63 bp 5′-untranslated region (UTR), and a 152 bp 3′-UTR with an AATAAAA frame and a 28-bp poly (A) tail (**Figure 2C**).

A neighbor-joining phylogenetic tree was constructed *via* the MEGA 6.0 software with 1,000 bootstrap replications, which based on alignment of the amino acid sequences of prePhHB and other amino acid sequences that encode the *Caryophyllaceae*-like CP precursors. Phylogenetic analysis showed that the 12 amino acid sequences were clustered into two branches: one branch was class I CPs of *Saponaria vaccaria* and CPs of *Dianthus caryophyllus*; another branch was included class II CPs of *Saponaria vaccaria* and HB of *P. heterophylla* (**Figure 2D**). Thus, prePhHB is likely the biosynthetic precursor of HB.

### *prePhHB* Gene Expression Is Positively Correlated With HB Accumulation in *P. Heterophylla*

**Figure 3:**
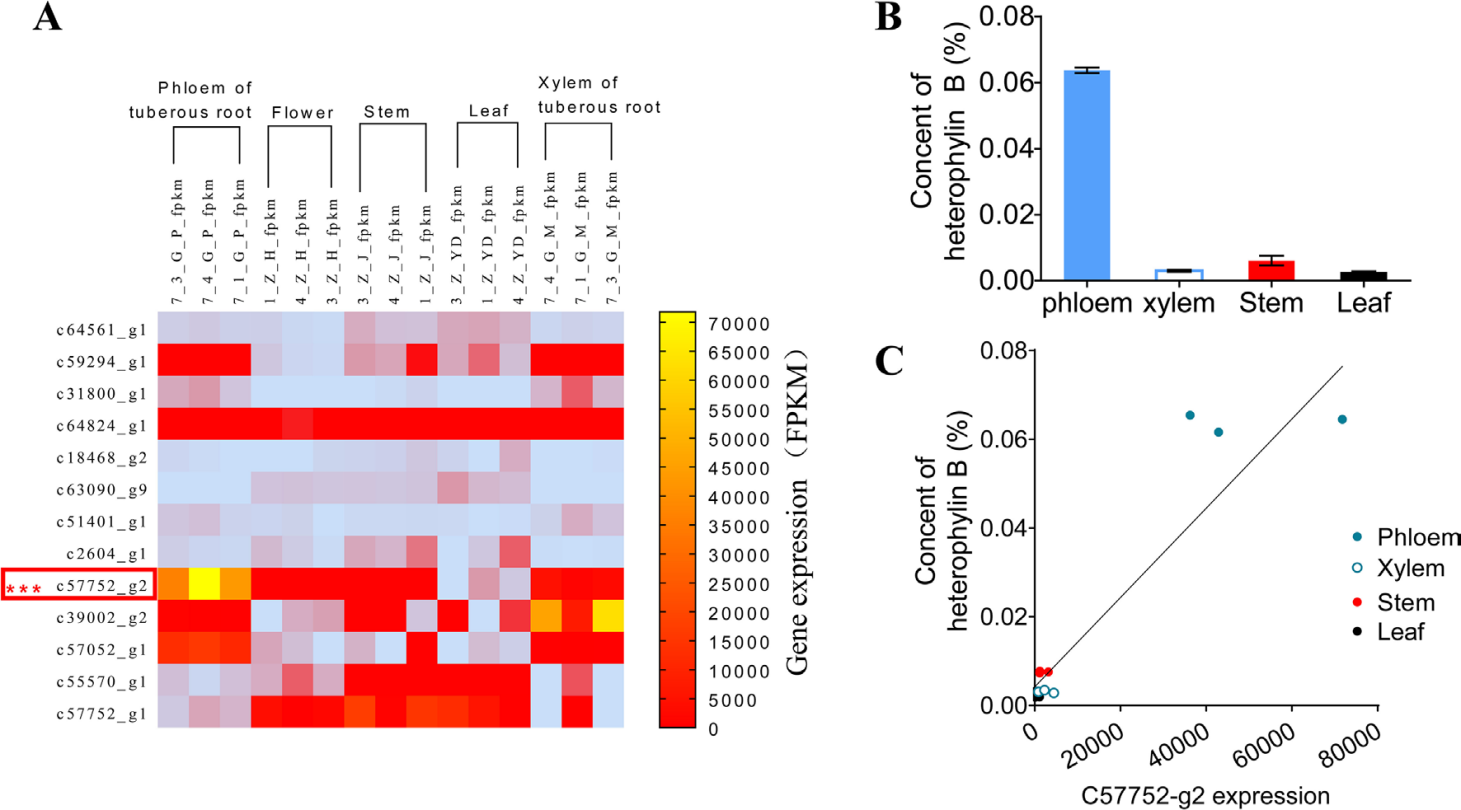
The relative expression level of *prePhHB* gene was positively correlated with the content of HB in the tissues of *P. heterophylla*. **(A)** Heat-map of the differentially expressed unigenes associated with biosynthesis of HB in phloem of tuberous roots, flower, stems, leaves, and xylem of tuberous roots of *P. heterophylla*. *prePhHB* is highlighted with a red box. Three biological replicates were plant numbers 1, 3, and 4, respectively (Li et al., 2016). **(B)** The content of HB in phloem of tuberous roots, stems, leaves, and xylem of tuberous roots of *P. heterophylla*. Bars represent the means values ± SEM of three biologically independent replicates. **(C)** Correlation between the relative expression level of *prePhHB* and the content of HB (i.e., Fig 3A *vs*. 3B), each independent point represents means of three technical replicates and the independent points, with same color, which indicate three biological replicates (r^2^ = 0.8768, p < 0.0001).

To preliminarily confirm the function of *prePhHB*, the expression levels of HB synthesis related genes were analyzed using FPKM (fragments per kilobase of exon model per million mapped reads) (**Figure 3A**) (Li et al., 2016). The results showed that the expression level of the *prePhHB* gene was highest among these related genes in the tuberous root. Furthermore, the *prePhHB* expression level in phloem of tuberous root surpassed xylem. We measured the HB content of four tissues of *P. heterophylla* and analyzed the correlation between *prePhHB* gene expression level and HB content. The results revealed that the HB content had a significant difference in these tissues (**Figure 3B**). The results of correlation analysis revealed that *prePhHB* expression level was positively correlated with the HB content in *P. heterophylla* (**Figure 3C**). Accordingly, the level of *prePhHB* transcription was completely in accordance with the HB accumulation pattern in the different organs of *P. heterophylla*, which further indicated that *prePhHB* is the precursor gene of HB.

### HB Is Derived From *prePhHB*-Encoded Linear Peptide in Enzymatic Conditions

**Figure 4:**
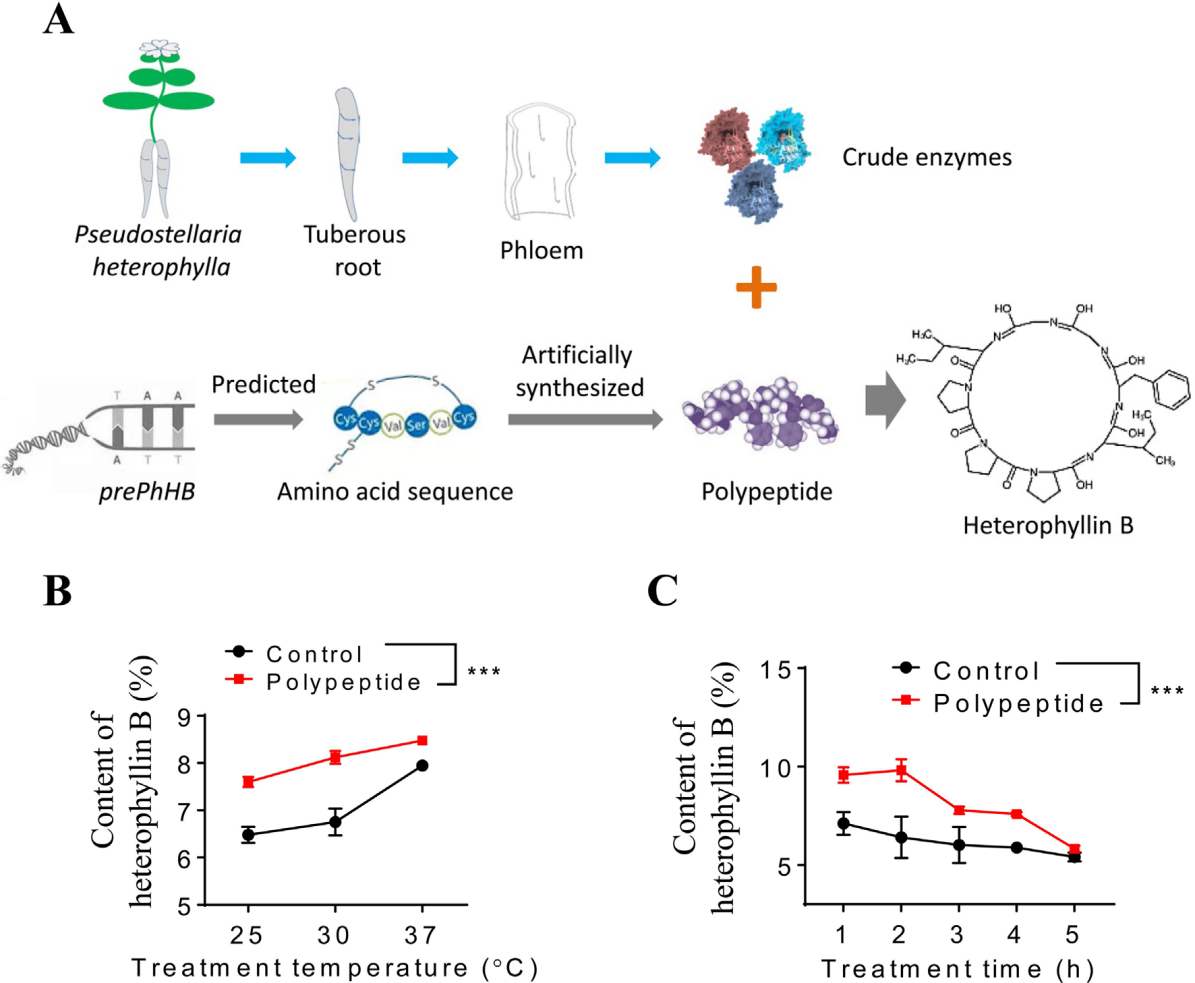
Enzymatic verification of the conversion of preheterophyllin B to HB *in vitro*. **(A)** Schematic diagram in which crude enzymes are combined with polypeptide in an aqueous environment to convert the substrate to HB through a series of reactions. **(B** and **C)** Quantitative analysis of the HB is formed by polypeptides under the action of crude enzymes *in vitro*, bars represent means ± SEM of four independent experiments, and asterisks represent significant difference between control and polypeptide, with a one-way ANOVA test (***p < 0.001).

To investigate whether the *prePhHB*-encoded linear peptide could be converted to HB, an enzyme-catalyzed reaction was tested *in vitro* (**Figure 4A**). As mentioned above, compared to other tissues of *P. heterophylla*, *prePhHB* was much more expressed in phloem of the tuberous roots; therefore, we hypothesized that the enzymes catalyzing the cyclization of the *prePhHB*-encoded linear peptides are mainly accumulated in developing phloem of the tuberous roots of *P. heterophylla*. Nevertheless, these enzymes and their corresponding functions have not been isolated and identified, respectively. Therefore, crude enzymes extracted from the phloem of tuberous roots of *P. heterophylla* were used in this experiment. The *prePhHB*-encoded linear peptide (MSTISAIHIMKPSIFGGLPPPSQELINGDDISLMV) was synthesized, and a purity of more than 86.71% and an MW of 1237.75 was confirmed *via* HPLC and LC-MS (**Figure S1**). The *prePhHB*-encoded linear peptide was catalyzed by crude enzymes for gradient time or temperature. The reaction products were also assayed for HB content *via* HPLC (**Figure S2**). The results showed that the content of HB was gradually increased with elevation of the reaction temperature, but it was gradually decreased with an increase of the reaction time. However, under different conditions, it is particularly noteworthy that the amount of produced cyclized products (HB) was significantly higher when compared with the control group (p < 0.001) (**Figures 4B, C**).

### Expression of the *prePhHB* in Tobacco Resulted in HB Generation

**Figure 5:**
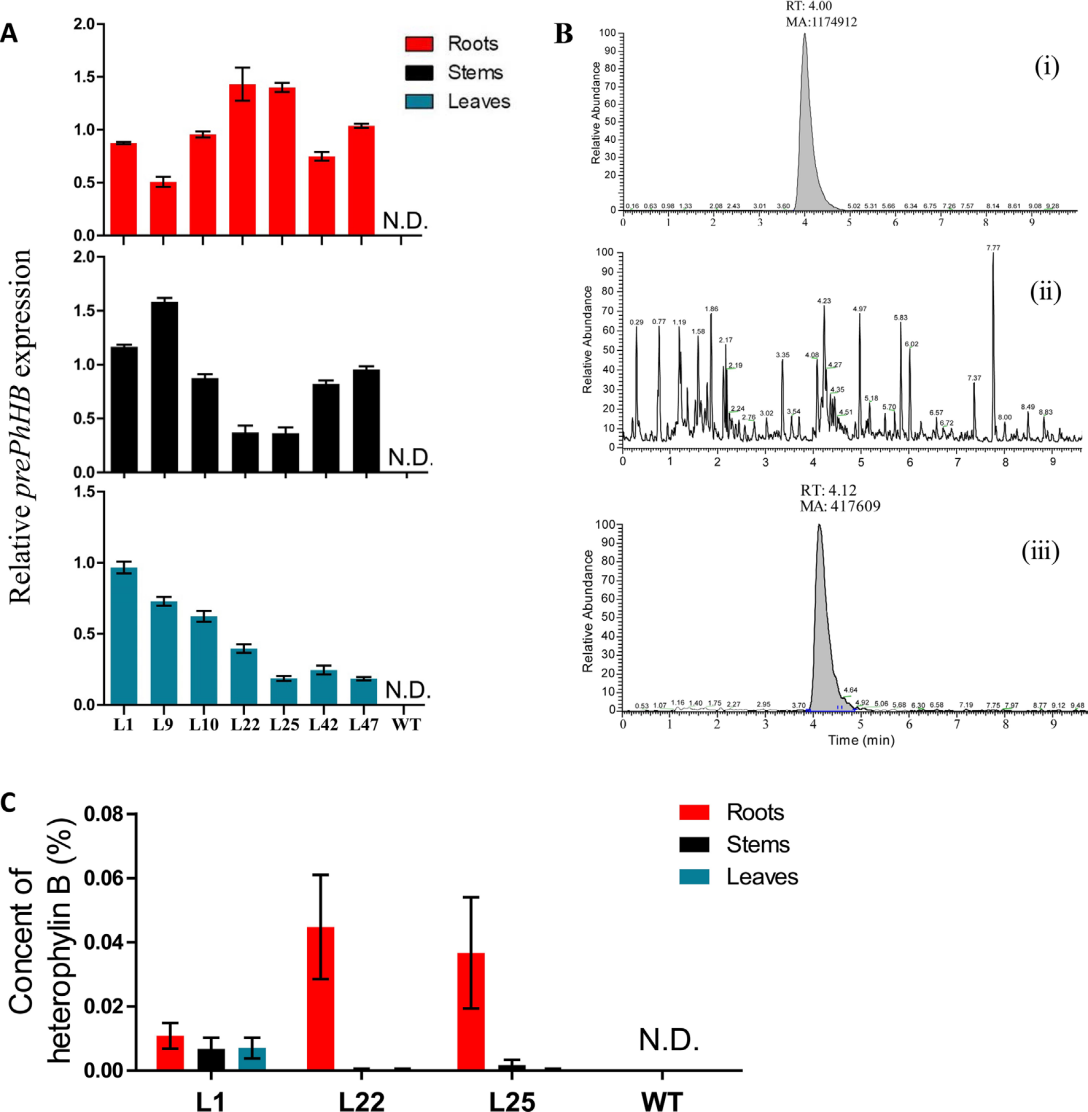
Recombinant expression of *prePhHB* in tobacco leaves enables HB production. **(A)** Quantification of the RT-PCR analysis results of the **Figure (S4B)** using ImageJ software. All analyses were performed with three biological replicates, and bars represent means ± SEM. **(B)** Total ion chromatograms (TIC) of the extracts of wild-type and transgenic tobacco are shown. (i) HB standard test *via* UPLC-MS/MS; (ii) no HB for WT tobacco was detected; (iii) expression of *prePhHB* leads to accumulation of HB (RT = 4.12 min). **(C)** Production of HB in transformed tobacco generated using *Agrobacterium* *tumefaciens* strain harboring pLGNL-*prePhHB* or WT tobacco. HB was determined by UHPLC-MS/MS. N.D. is not detected, and data are the means ± SEM of three biological replicates.

To further identify the products of *prePhHB*, the *prePhHB* gene was introduced into tobacco using *Agrobacterium*-mediated transformation. The full-length coding sequence of *prePhHB* was cloned into the pLGNL expression vector and was genetically transformed in tobacco leaves. After co-cultivation for 3 days in MS medium, the plants were transferred to a selection medium with 100 mg/L Kan and 500 mg/L Cef. The kanamycin-resistant plants and PCR positive plants were transferred to the rooting medium (**Figures S3** and **S4A**). Next, the plants with complete root development were selected and transferred to a greenhouse for further growth. Total RNA was extracted from the stems, leaves, roots of transgenic lines, and WT plants and subjected to RT-PCR using specific primers of *prePhHB*. As expected, expression of *prePhHB* was detected in the stems, leaves, and roots of transgenic plants but was not detected in WT tobacco (**Figures 5A** and **S4B**).

To determine whether the expression of *prePhHB* enables HB formation, the metabolites were extracted and subjected to UPLC-MS/MS analysis. Based on UPLC-MS/MS, the roots, stems, and leaves of transgenic lines (L1, L22 and L25) were found to contain HB, while no HB was detected in the roots, stems, and leaves of WT tobacco (**Figure 5B**). Moreover, the HB content in L22 roots was the highest, up to 0.044% (**Figure 5C**). Thus, HB is derived from the precursor gene *prePhHB*.

### Mutations in the *prePhHB* Gene May Affect the Accumulation of HB in “FJZR”

**Figure 6:**
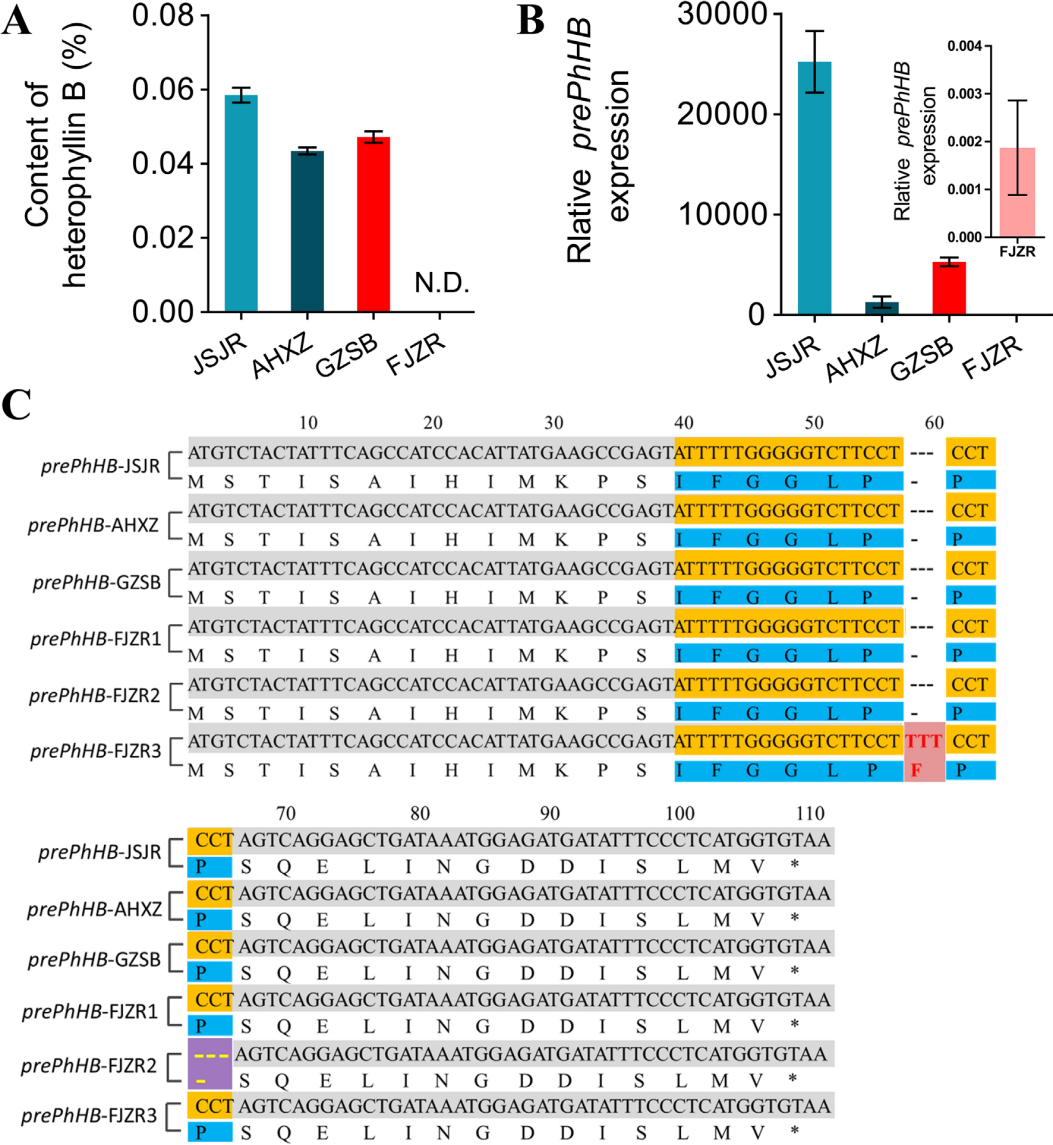
Mutations of *prePhHB* may result in accumulation of HB in “FJZR”. **(A)** HB was identified and quantified using HPLC-DAD, and N.D. is not detected. Bars represent the mean values ± SEM of three biological replicates. **(B)** The expression pattern of *prePhHB* in tuberous roots of *P. heterophylla* that were originated in different regions: “JSJR”, “FJZR”, “AHXZ”, and “GZSB”, respectively, using RT-qPCR. The values are the means ± SEM of three biological replicates. **(C)** The sequence alignment of *prePhHB* among *P.* *heterophylla* from the “JSJR”, “FJZR”, “AHXZ”, and “GZSB”. Mutation sites were highlighted by striking color. Red areas represent insertion mutation, and purple areas represent deletion mutation.

To investigate the mechanisms underlying regional difference in the HB content in *P. heterophylla* from different regions, we transplanted *P. heterophylla* from different regions (Jiangsu, Anhui, Fujian, Guizhou) to the same place (Guizhou) for 1 year to eliminate environmental factors. HB was identified and quantified using HPLC-DAD. We found that there were significant differences in the HB content among four provenances (“JSJR”, “FJZR”, “AHXZ”, and “GZSB”). However, HB was not detected in the tuberous roots of “FJZR” (**Figure 6A**). The expression level of *prePhHB* was evaluated *via* RT-qPCR. As shown in **Figure 6B**, the *prePhHB* was low expression in “FJZR”, which is consistent with the change in HB content.

To further explore the molecular mechanism of low expression of the *prePhHB* in “FJZR”, we cloned and sequenced the full-length coding sequence of *prePhHB* from “JSJR”, “FJZR”, “AHXZ”, and “GZSB”. The DNA sequencing results showed that two mutations were detected in “FJZR” in addition to the normal *prePhHB-*FJZR1 gene of the *prePhHB*. Concretely, three pairs of bases were inserted into 54 to 56 sites in the ORF of the *prePhHB-*FJZR2, which resulted in the increase of 1 amino acid residue (Phe). Meanwhile, three pairs of bases were deleted at 61 to 63 in the ORF of the *prePhHB-*FJZR3, which resulted in the deletion of one amino acid residue (Pro) (**Figure 6C**).

## Discussion

*Caryophyllaceae*-type CPs have been reported from *Caryophyllaceae* family, *Rhamnaceae* family, and other eight families (Picur et al., 2006; Cascales and Craik, 2010). Although evidence has been presented for biosynthesis of segetalins in *Saponaria vaccaria L*., very little known is the biosynthesis of CPs in *P. heterophylla* (Condie et al., 2011). In the present study, we developed a new method to screen the precursor genes of HB in *P. heterophylla,* which will provide new ideas for the study of the *Caryophyllaceae*-type CPs with known chemical structures, but their core peptides and precursor genes are unknown. Specifically, this method is to take 16 possible amino acid sequences after ring-opening of the cyclic octapeptide HB as query sequences and screen them from six-frame translations of the *P. heterophylla* RNA-seq database to accurately match the candidate genes (**Figure 2A**).

From the transcriptome data, we found that only 1 (IFGGLPPP) of the 16 amino acid sequences was perfectly matched. The deduced protein sequence of this sequence (prePhHB) shows high similarity to *Caryophyllaceae*-like CP precursor peptides. Moreover, in the phylogenetic tree, the prePhHB is closely related to the precursor peptides of segetalin F and segetalin J of *Saponaria vaccaria*. These results suggest that the prePhHB may be the precursor peptide of HB. Most interestingly, prePhHB contained a core peptide (IFGGLPPP) motif. However, this is different from previous reports that the core peptide of HB may be GGLPPPIF (Xu et al., 2012). In this study, we identified, cloned, and characterized the *prePhHB* from the tuberous root of *P. heterophylla via* RNA-seq database and the RACE method. The *prePhHB* function was confirmed through an *in vitro* enzyme assay of the crude enzymes obtained from the phloem of tuberous roots of *P. heterophylla* and was demonstrated *via* heterologous expression of this gene in tobacco and analysis of HB in extracts. These results demonstrated that HB is ribosome-dependent production derived from the precursor peptide prePhHB, and plant genomes do not encode NRPSs (Kersten and Weng, 2018). Therefore, we think that HB is ribosomally synthesized and posttranslationally modified peptide (RiPP). Additionally, HB can be produced in tobacco when the *prePhHB* gene is present and expressed. It suggests that tobacco must contain enzymes necessary to process the precursor peptide to engender HB. Interestingly, similar results were also found in lyciumin A, B, and D (i.e., three plant CPs) (Kersten and Weng, 2018).

Besides, we discovered the existence of significant differences in HB content in cultivated *P. heterophylla* from different areas. The content of HB was the highest in the Jiangsu *P. heterophylla*; however, almost no HB was detected in the Fujian *P. heterophylla*. Previous studies have also shown that there are obvious differences in the quality of different cultivated *P. heterophylla* from different fields (Hua et al., 2016b; Hua et al., 2016c). These variances might be related to differences in the ecological environment, genetic information, and cultivation techniques. To eliminate the effect of environmental factors on HB accumulation in *P. heterophylla*, we transplanted *P. heterophylla* seedling from four provinces (Fujian, Jiangsu, Anhui, and Guizhou) to the same environment. We found that the content of HB also could not detect in the *P. heterophylla* which transplanted from Fujian province. These data suggested that the difference in content of HB mainly related to the germplasm genetic factors.

Given this interesting phenomena, under the premise of eliminating the ecological environment and cultivation technology, we established correlations between *prePhHB* expression patterns and HB distributions in “JSJR”, ‘FJZR”, “AHXZ”, and “GZSB”. Interestingly, the relative expression level of *prePhHB* among “JSJR”, “AHXZ”, and “GZSB” that the differences among cultivars were very high but not much different in HB content. It is suggested that the formation and accumulation of HB in *P. heterophylla* may be influenced by the precursor gene *prePhHB*, the key enzyme genes of biosynthesis, and other regulatory genes. More interestingly, we found that there are two mutants of *prePhHB* in “FJZR,” *prePhHB*-FJZR2 and *prePhHB*-FJZR3, respectively, as well as normal *prePhHB*-FJZR1, but HB has not been detected. This result can be explained as follows: although *prePhHB*-FJZR1 contains no mutation, the relative expression of the *prePhHB*-FJZR1 gene is less than 0.002, so the amount of HB formed may be very small, which is less than the minimum detection limit of HPLC analysis. And under the chromatographic conditions of this study, the minimum detection limit of HB was 0.673 µg/ml. Moreover, *Caryophyllaceae*-like CPs are head-to-tail-cyclized plant CPs (Spengler et al., 2005; Arnison et al., 2013). Thus, the mutant genes *prePhHB*-FJZR2 and *prePhHB*-FJZR3 may encode two new *Caryophyllaceae*-like CPs: cyclo-[IFGGLPP] and cyclo-[IFGGLPFPP], which may be detected in the crude extracts of FJZR samples *via* the UPLC-MS-MS. These results indicate that the mutation of *prePhHB* leads to the formation of an abnormal linear peptide in the precursors of HB, which ultimately affects the synthesis of HB. Further UPLC-MS-MS analysis and NMR analysis experiments would be needed to draw conclusions about the influence formation of CPs that the changes of amino acids in *prePhHB*-FJZR2 and *prePhHB*-FJZR3.

Overall, our results suggest that HB is RiPP derived from the precursor gene *prePhHB*-encoded precursor peptide, and the core peptide sequence of HB is IFGGLPPP in *P. heterophylla*. These findings developed a new idea for the rapid identification of *Caryophyllaceae*-type CP precursor peptides *via* RNA-seq data mining. Further work should characterize the enzymes involved in the biosynthesis of HB.

## Data Availability Statement

The coding sequence for *prePhHB* can be found in GenBank, using accession number MH699110.

## Author Contributions

WZ, TZ, JL, and WJ planned and designed the research. WZ, JL, CY, and RX performed the experiments and analyzed the data. WZ, TZ, JZ, and WJ wrote the manuscript. CX, DW, CZ, AG, and YB contributed equally to this work.

## Funding

This research was supported by the National Natural Science Foundation of China (Grant No.81460579), High-level Innovative Talents of Guizhou Province of China (Qian Ke He Platform and Talent [2018]5638), Science and Technology Project in Guizhou Province of China (Qian Ke He Platform and Talent [2019]5611), Key project at central government level: The ability establishment of sustainable use for valuable Chinese medicine resources (Grant No. 2060302) and First-class Discipline Construction Projects of Guizhou Province of China (GNYL(2017)008).

## Conflict of Interest

The authors declare that the research was conducted in the absence of any commercial or financial relationships that could be construed as a potential conflict of interest.

## Supplementary Material

The Supplementary Material for this article can be found at: https://www.frontiersin.org/articles/10.3389/fpls.2019.01259/full#supplementary-material

Figure S1 Supplementary information for **Figure 4**. Identification of *prePhHB*-encoded linear peptide *via* HPLC and LC-MS. **(A)** Purity testing via HPLC. **(B)** Molecular weight detection *via* LC-MS.

Figure S2 Supplementary information for **Figure 4**. HPLC chromatograms showing products of reactions with the conversion of preheterophyllin B (linear polypeptide) to HB (cyclic peptide) *in vitro*. (i) HB standard; (ii) Control group, no polypeptide was added; (iii) Polypeptide group (mAU, milli-absorbance unit; the unit of retention time is min).

Figure S3 PCR amplification of the full-length coding sequence of *prePhHB* from genomic DNA extracted from transgenic tobacco. **(A)** PCR amplification using specific primers for the *prePhHB* gene. **(B)** PCR amplification using specific primers for the plant expression vector PLGNL. The left M is 5000 bp Marker and the right M is 500 bp Marker; CK1: Wild-type tobacco; CK2: PLGNL-*prePhHB* plasmid DNA; CK3: LBA4404-PLGNL-*prePhHB* bacteria liquid; Numbers 1 to 50: transgenic tobacco plants.

Figure S4 Supplementary information for **Figure 5**. **(A)** Kanamycin sensitivity tests that the above picture is kanamycin-resistant plants and the following picture is albino plants. **(B)** RT-PCR analysis of relative mRNA levels of *prePhHB* and *ACTIN* in stems, leaves, and roots of transgenic and wild-type plants. *ACTIN* gene was used as an internal control. Transgenic lines (L1, L9, L10, L22, L25, L42 and L47) were transformed by Agrobacterium-mediated transformation containing the pLGNL-*prePhHB*.

## References

[B1] Arnison P. G. Bibb M. J. Bierbaum G. Bowers A. A. Bugni T. S. Bulaj G. (2013). Ribosomally synthesized and post-translationally modified peptide natural products: overview and recommendations for a universal nomenclature. Nat. Prod. Rep. 30, 108–160. 10.1039/C2NP20085F 23165928 PMC3954855

[B2] Arseneau J. R. Steeves R. Laflamme M. (2017). Modified low-salt CTAB extraction of high-quality DNA from contaminant-rich tissues. Mol. Ecol. Resour. 17, 686–693. 10.1111/1755-0998.12616 27768249

[B3] Ballester M. Cordon R. Folch J. M. (2013). DAG expression: high-throughput gene expression analysis of real-time PCR data using standard curves for relative quantification. PLoS One 8, e80385. 10.1371/journal.pone.0080385 24260380 PMC3832397

[B4] Bionda N. Fasan R. (2017). Ribosomal synthesis of thioether-bridged bicyclic peptides. Methods Mol. Biol. 1495, 57–76. 10.1007/978-1-4939-6451-2_5 27714610 PMC5777217

[B5] Burman R. Gruber C. W. Rizzardi K. Herrmann A. Craik D. J. Gupta M. P. (2010). Cyclotide proteins and precursors from the genus Gloeospermum: filling a blank spot in the cyclotide map of Violaceae. Phytochemistry 71, 13–20. 10.1016/j.phytochem.2009.09.023 19879608

[B6] Burman R. Gunasekera S. Stromstedt A. A. Goransson U. (2014). Chemistry and biology of cyclotides: circular plant peptides outside the box. J. Nat. Prod. 77, 724–736. 10.1021/np401055j 24527877

[B7] Cai Y. Zhang C. Hao L. Chen J. Xie P. Chen Z. (2016). Systematic identification of seven ribosomal protein genes in bighead carp and their expression in response to microcystin-LR. J. Toxicol. Sci. 41, 293–302. 10.2131/jts.41.293 26961614

[B8] Cascales L. Craik D. J. (2010). Naturally occurring circular proteins: distribution, biosynthesis and evolution. Org. Biomol. Chem. 8, 5035–5047. 10.1039/c0ob00139b 20835453

[B9] Condie J. A. Nowak G. Reed D. W. Balsevich J. J. Reaney M. J. Arnison P. G. (2011). The biosynthesis of Caryophyllaceae-like cyclic peptides in Saponaria vaccaria L. from DNA-encoded precursors. Plant. J. 67, 682–690. 10.1111/j.1365-313X.2011.04626.x 21554452

[B10] Daly N. L. Chen Y. K. Foley F. M. Bansal P. S. Bharathi R. Clark R. J. (2006). The absolute structural requirement for a proline in the P3’-position of Bowman-Birk protease inhibitors is surmounted in the minimized SFTI-1 scaffold. J. Biol. Chem. 281, 23668–23675. 10.1074/jbc.M601426200 16766795

[B11] Finking R. Marahiel M. A. (2004). Biosynthesis of nonribosomal peptides1 . Annu. Rev. Microbiol. 58, 453–488. 10.1146/annurev.micro.58.030603.123615 15487945

[B12] Gillon A. D. Saska I. Jennings C. V. Guarino R. F. Craik D. J. Anderson M. A. (2008). Biosynthesis of circular proteins in plants. Plant. J. 53, 505–515. 10.1111/j.1365-313X.2007.03357.x 18086282

[B13] Han C. Shen Y. Chen J. Lee F. S. Wang X. (2008). HPLC fingerprinting and LC-TOF-MS analysis of the extract of Pseudostellaria heterophylla (Miq.) Pax root. J. Chromatogr. B Analyt. Technol. Biomed. Life Sci. 862, 125–131. 10.1016/j.jchromb.2007.11.041 18155974

[B14] Henriques S. T. Huang Y. H. Rosengren K. J. Franquelim H. G. Carvalho F. A. Johnson A. (2011). Decoding the membrane activity of the cyclotide kalata B1: the importance of phosphatidylethanolamine phospholipids and lipid organization on hemolytic and anti-HIV activities. J. Biol. Chem. 286, 24231–24241. 10.1074/jbc.M111.253393 21576247 PMC3129204

[B15] Hu J. Pang W. Chen J. Bai S. Zheng Z. Wu X. (2013). Hypoglycemic effect of polysaccharides with different molecular weight of Pseudostellaria heterophylla. BMC Complement Altern. Med. 13, 267. 10.1186/1472-6882-13-267 24131482 PMC3853478

[B16] Hua Y. Hou Y. Wang S. Ma Y. Liu Z. Zou L. (2016a). Comparison of chemical compositions in pseudostellariae radix from different cultivated fields and germplasms by NMR-based metabolomics. Molecules 21 (11), 1538. 10.3390/molecules21111538 PMC6273876 27854294

[B17] Hua Y. Wang S. Chai C. Liu Z. Liu X. Zou L. (2016b). Quality evaluation of pseudostellariae radix based on simultaneous determination of multiple bioactive components combined with grey relational analysis. Molecules 22 (10): E13. 10.3390/molecules22010013 28035970 PMC6155879

[B18] Hua Y. Wang S. Liu Z. Liu X. Zou L. Gu W. (2016c). Transcriptomic analysis of Pseudostellariae Radix from different fields using RNA-seq. Gene 588, 7–18. 10.1016/j.gene.2016.04.043 27125225

[B19] Jhamandas J. H. Goncharuk V. (2013). Role of neuropeptide FF in central cardiovascular and neuroendocrine regulation. Front. Endocrinol. (Lausanne) 4, 8. 10.3389/fendo.2013.00008 23404625 PMC3566396

[B20] Jia A. Xiang L. I. Tan N. Liu X. Shen Y. Zhou J. (2006). Enzymatic cyclization of linear peptide to plant cyclopeptide heterophyllin B. Sci. China Ser. B. 49, 63–66. 10.1007/s11426-005-0204-5

[B21] Kersten R. D. Weng J. K. (2018). Gene-guided discovery and engineering of branched cyclic peptides in plants. Proc. Natl. Acad. Sci. U. S. A. 115, E10961–E10969. 10.1073/pnas.1813993115 30373830 PMC6243238

[B22] Li J. Zheng W. Long D. Ding L. Gong A. Xiao C. (2016). De novo sequencing and assembly analysis of the pseudostellaria heterophylla transcriptome. PLoS One. 11, e0164235. 10.1371/journal.pone.0164235 27764127 PMC5072632

[B23] Luo H. Hallen-Adams H. E. Scott-Craig J. S. Walton J. D. (2012). Ribosomal biosynthesis of α-amanitin in Galerina marginata. Fungal Genet. Biol. 49, 123–129. 10.1016/j.fgb.2011.12.005 22202811 PMC3997167

[B24] Morita H. Kayashita T. Takeya K. Itokawa H. (1995). Cyclic peptides from higher plants, Part 15. Pseudostellarin H, a new cyclic octapeptide from Pseudostellaria heterophylla. J. Nat. Prod. 58, 943–947. 10.1016/0012-821X(88)90038-6 7673942

[B25] Noh H. J. Hwang D. Lee E. S. Hyun J. W. Yi P. H. Kim G. S. (2015). Anti-inflammatory activity of a new cyclic peptide, citrusin XI, isolated from the fruits of Citrus unshiu. J. Ethnopharmacol. 163, 106–112. 10.1016/j.jep.2015.01.024 25625351

[B26] Otzen D. E. (2017). Biosurfactants and surfactants interacting with membranes and proteins: same but different? Biochim. Biophys. Acta 1859, 639–649. 10.1016/j.bbamem.2016.09.024 27693345

[B27] Pandey M. B. Singh S. Malhotra M. Pandey V. B. Singh T. D. (2012). Two new 14-membered cyclopeptide alkaloids from Zizyphus xylopyra. Nat. Prod. Res. 26, 836–841. 10.1080/14786419.2011.559641 21995325

[B28] Pang W. Lin S. Dai Q. Zhang H. Hu J. (2011). Antitussive activity of Pseudostellaria heterophylla (Miq.) Pax extracts and improvement in lung function *via* adjustment of multi-cytokine levels. Molecules 16, 3360–3370. 10.3390/molecules16043360 21512444 PMC6260644

[B29] Pathi K. M. Tula S. Tuteja N. (2013). High frequency regeneration *via* direct somatic embryogenesis and efficient Agrobacterium-mediated genetic transformation of tobacco. Plant Signal Behav. 8, e24354. 10.4161/psb.24354 23518589 PMC3906319

[B30] Picur B. Cebrat M. Zabrocki J. Siemion I. Z. (2006). Cyclopeptides of Linum usitatissimum. J. Pept. Sci. 12, 569–574. 10.1002/psc.779 16878298

[B31] Raju R. Gromyko O. Andriy B. Fedorenko V. Luzhetskyy A. Muller R. (2014). Oleamycins A and B: new antibacterial cyclic hexadepsipeptides isolated from a terrestrial Streptomyces sp. J. Antibiot. (Tokyo) 67, 339–343. 10.1038/ja.2014.1 24448627

[B32] Rodriguez-Vazquez N. Ozores H. L. Guerra A. Gonzalez-Freire E. Fuertes A. Panciera M. (2014). Membrane-targeted self-assembling cyclic peptide nanotubes. Curr. Top Med. Chem. 14, 2647–2661. 10.2174/1568026614666141215143431 25515753

[B33] Schmidt E. W. Nelson J. T. Rasko D. A. Sudek S. Eisen J. A. Haygood M. G. (2005). Patellamide A and C biosynthesis by a microcin-like pathway in Prochloron didemni, the cyanobacterial symbiont of Lissoclinum patella. Proc. Natl. Acad. Sci. U. S. A. 102, 7315–7320. 10.1073/pnas.0501424102 15883371 PMC1091749

[B34] Spengler J. Jimenez J. C. Burger K. Giralt E. Albericio F. (2005). Abbreviated nomenclature for cyclic and branched homo- and hetero-detic peptides. J. Pept. Res. 65, 550–555. 10.1111/j.1399-3011.2005.00254.x 15885114

[B35] Tan N. H. Zhou J. (2006). Plant cyclopeptides. Chem. Rev. 106, 840–895. 10.1021/cr040699h 16522011

[B36] Tantai J. C. Zhang Y. Zhao H. (2016). Heterophyllin B inhibits the adhesion and invasion of ECA-109 human esophageal carcinoma cells by targeting PI3K/AKT/beta-catenin signaling. Mol. Med. Rep. 13, 1097–1104. 10.3892/mmr.2015.4659 26647768 PMC4732845

[B37] Walton J. D. Hallen-Adams H. E. Luo H. (2010). Ribosomal biosynthesis of the cyclic peptide toxins of Amanita mushrooms. Biopolymers 94, 659–664. 10.1002/bip.21416 20564017 PMC4001729

[B38] Wang Z. Liao S. G. He Y. Li J. Zhong R. F. He X. (2013). Protective effects of fractions from Pseudostellaria heterophylla against cobalt chloride-induced hypoxic injury in H9c2 cell. J. Ethnopharmacol. 147, 540–545. 10.1016/j.jep.2013.03.053 23542142

[B39] Xu W. Zhu H. Tan N. Tang J. Zhang Y. Cerny R. L. (2012). An *in vitro* system to study cyclopeptide heterophyllin B biosynthesis in the medicinal plant Pseudostellaria heterophylla. Plant Cell Tissue Organ 108, 137–145. 10.1007/s11240-011-0022-8

[B40] Yang C. You L. Yin X. Liu Y. Leng X. Wang W. (2018). Heterophyllin B ameliorates lipopolysaccharide-induced inflammation and oxidative stress in RAW 264.7 Macrophages by Suppressing the PI3K/Akt Pathways. Molecules 23 (4): E717. 10.3390/molecules23040717 29561811 PMC6017815

[B41] Zhao W. O. Pang L. Dong N. Yang S. (2015). LC-ESI-MS/MS analysis and pharmacokinetics of heterophyllin B, a cyclic octapeptide from Pseudostellaria heterophylla in rat plasma. Biomed. Chromatogr. 29, 1693–1699. 10.1002/bmc.3481 25967583

