# Supplementary figures and images for: The Biosynthesis of Heterophyllin B in Pseudostellaria heterophylla From prePhHB-Encoded Precursor
Source: Front Plant Sci. 2019 Oct 17;10:1259. doi: 10.3389/fpls.2019.01259 (PMC6842982; doi:10.3389/fpls.2019.01259)

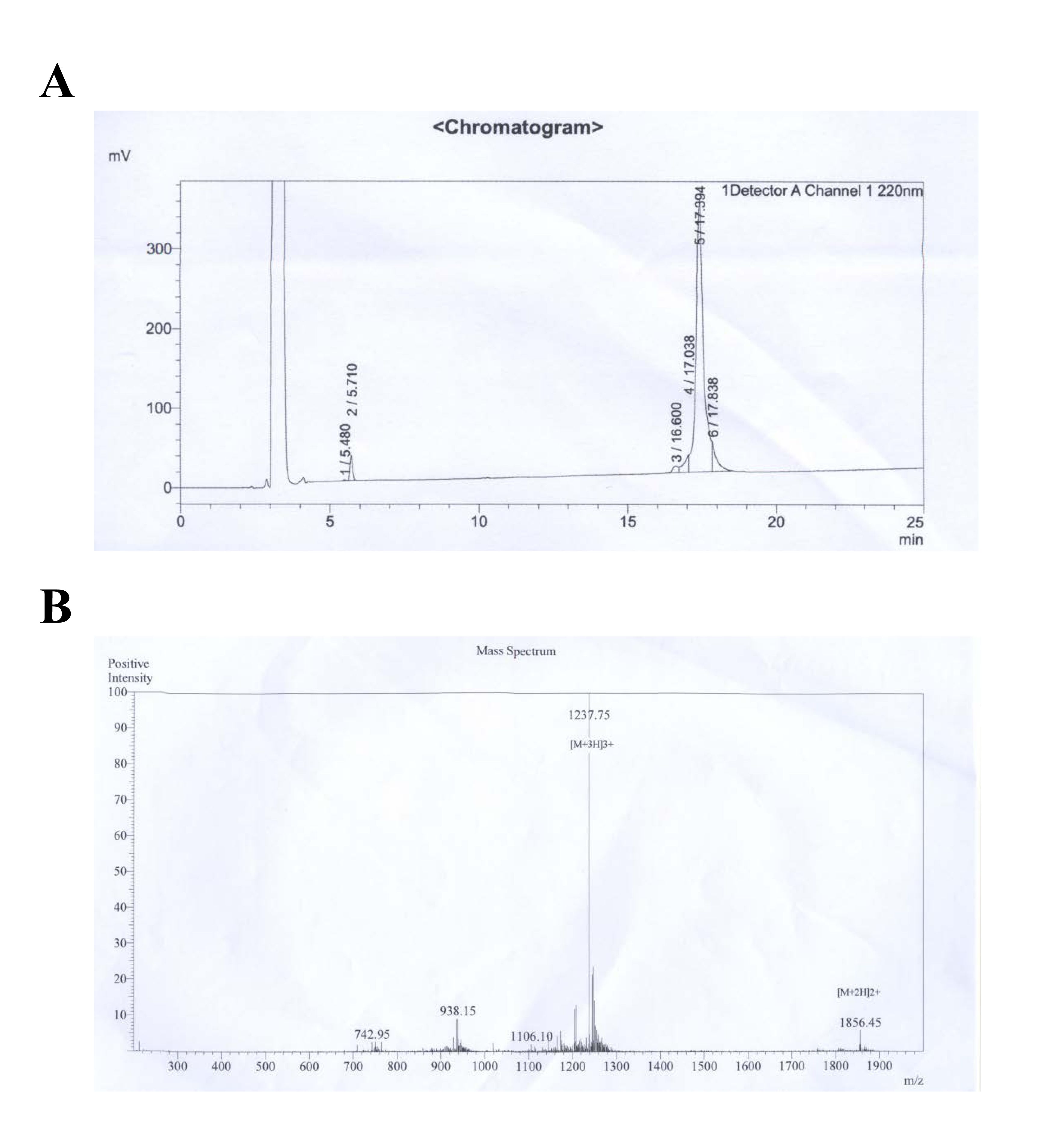

Supplement: Figure S1 — Supplementary information for Figure 4 . Identification of prePhHB-encoded linear peptide via HPLC and LC-MS. (A) Purity testing via HPLC. (B) Molecular weight detection via LC-MS. [file Image_1.jpeg]

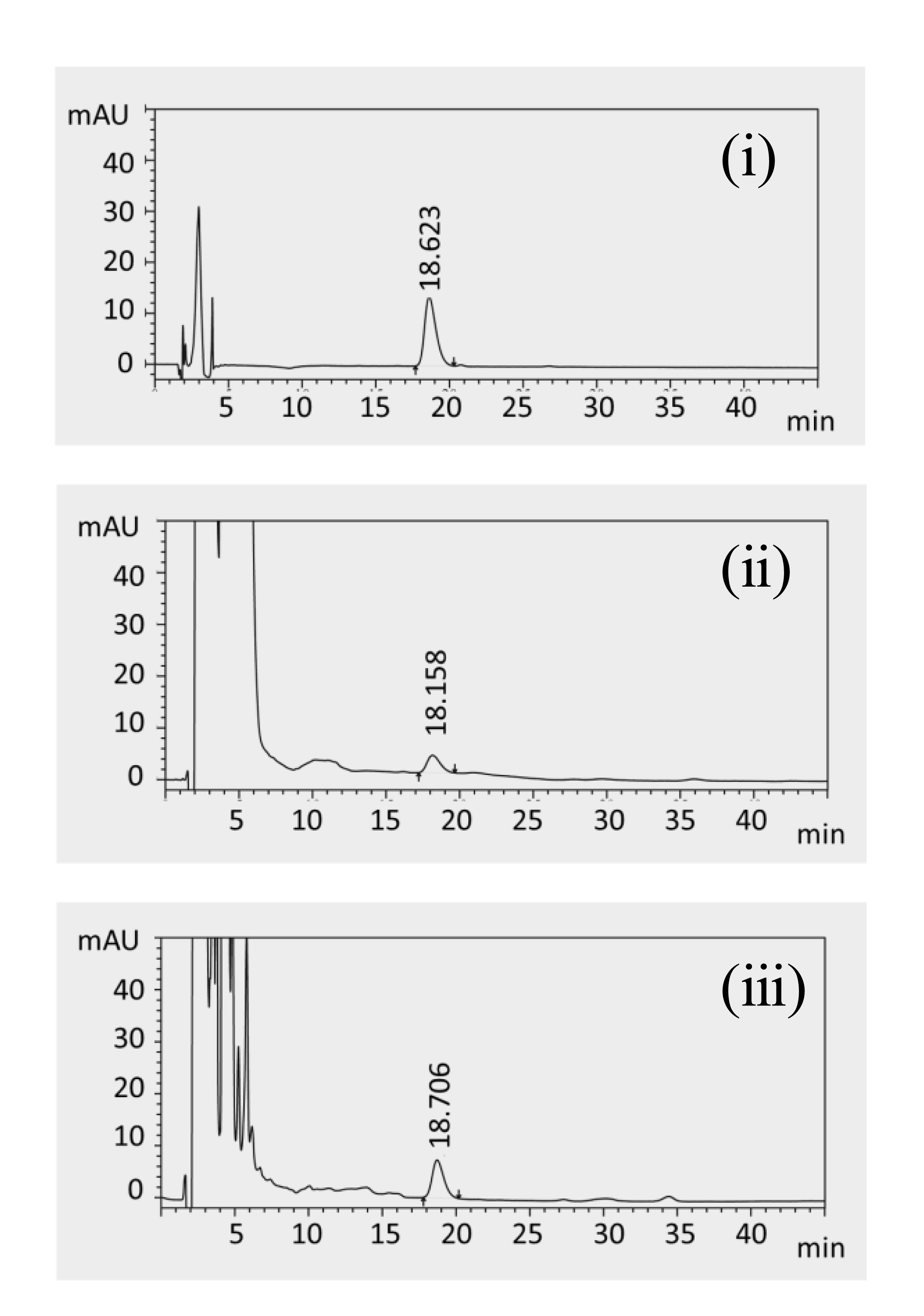

Supplement: Figure S2 — Supplementary information for Figure 4 . HPLC chromatograms showing products of reactions with the conversion of preheterophyllin B (linear polypeptide) to HB (cyclic peptide) in vitro. (i) HB standard; (ii) Control group, no polypeptide was added; (iii) Polypeptide group (mAU, milli-absorbance unit; the unit of retention time is min). [file Image_2.jpeg]

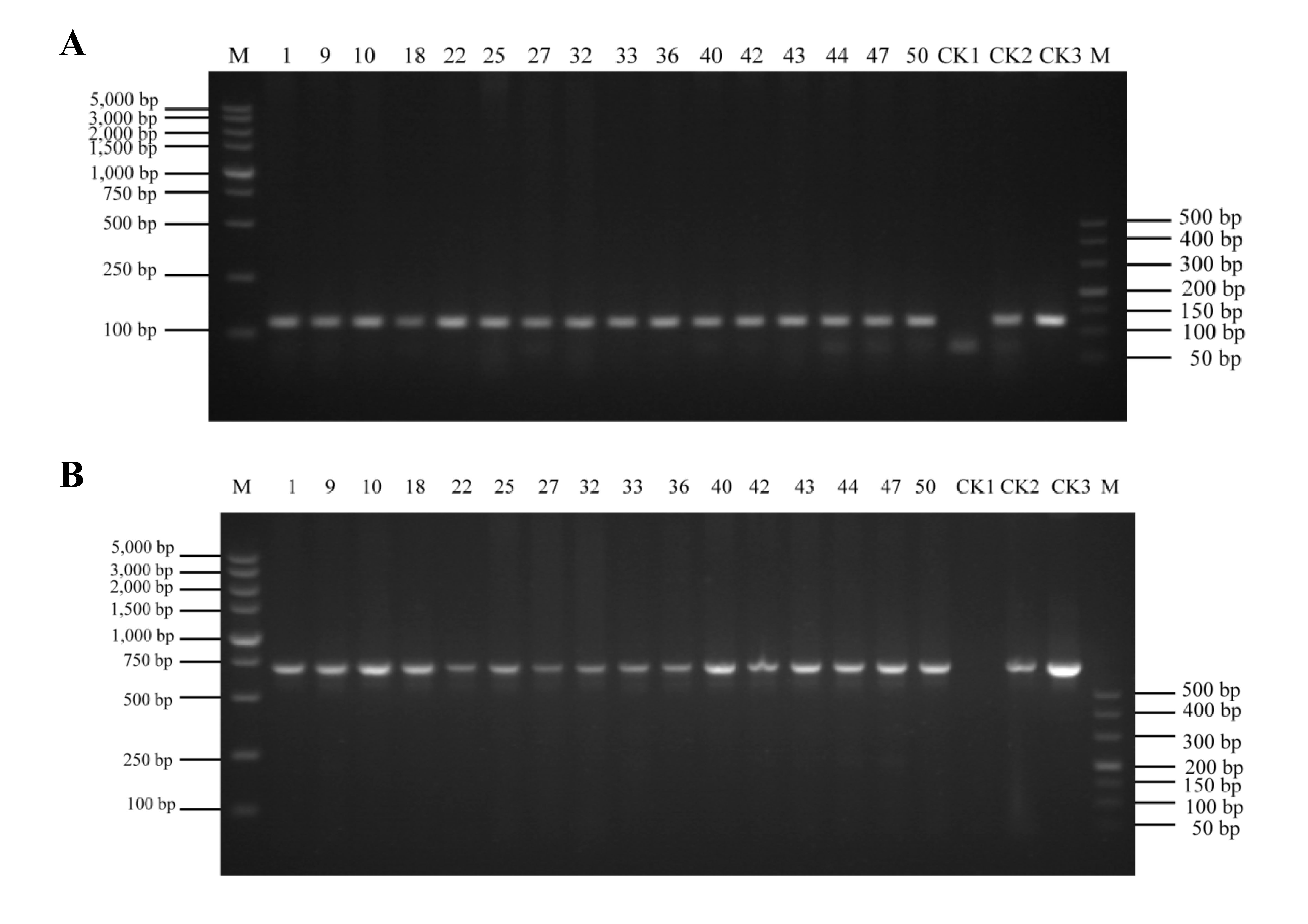

Supplement: Figure S3 — PCR amplification of the full-length coding sequence of prePhHB from genomic DNA extracted from transgenic tobacco. (A) PCR amplification using specific primers for the prePhHB gene. (B) PCR amplification using specific primers for the plant expression vector PLGNL. The left M is 5000 bp Marker and the right M is 500 bp Marker; CK1: Wild-type tobacco; CK2: PLGNL-prePhHB plasmid DNA; CK3: LBA4404-PLGNL-prePhHB bacteria liquid; Numbers 1 to 50: transgenic tobacco plants. [file Image_3.jpeg]

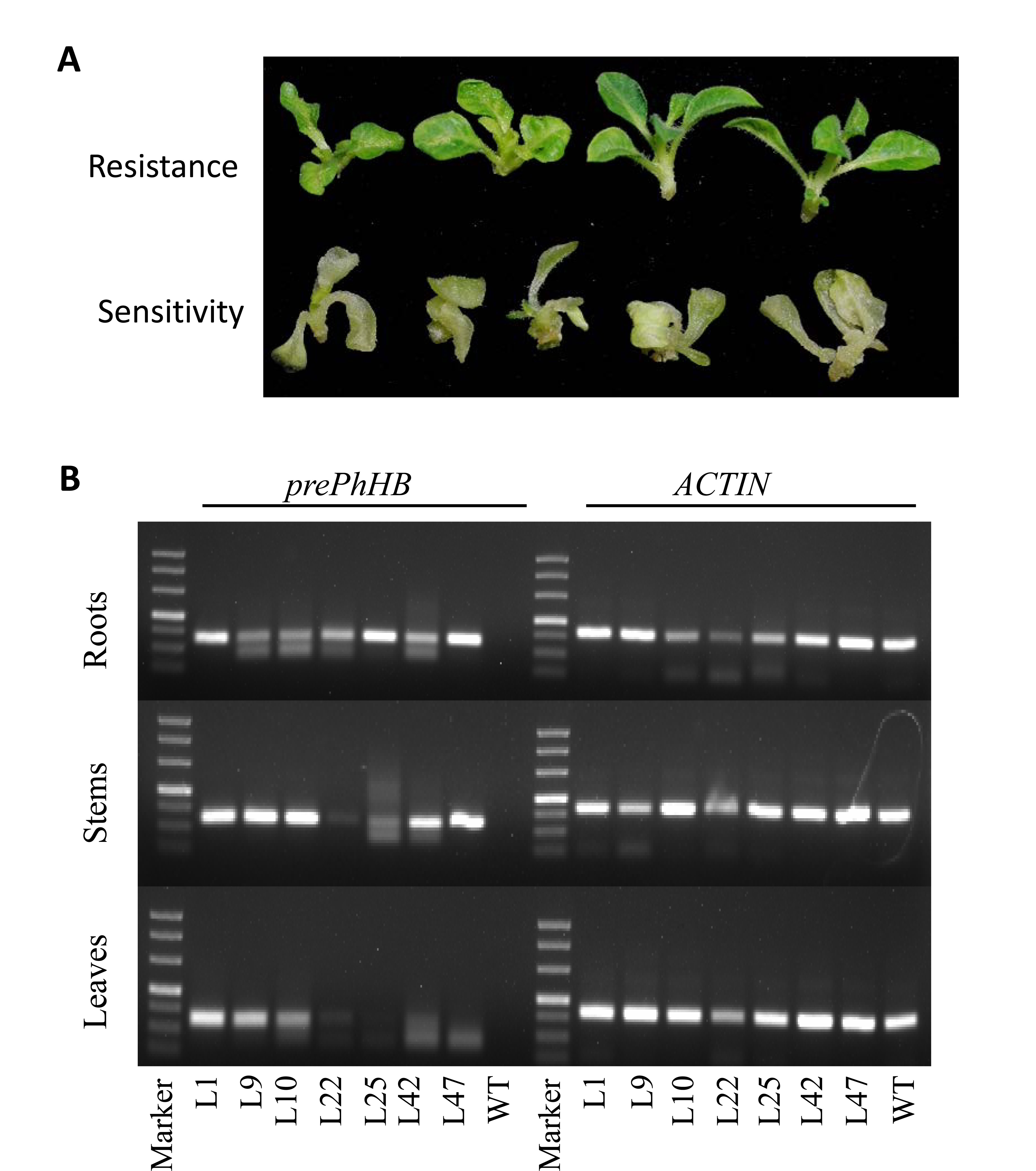

Supplement: Figure S4 — Supplementary information for Figure 5 . (A) Kanamycin sensitivity tests that the above picture is kanamycin-resistant plants and the following picture is albino plants. (B) RT-PCR analysis of relative mRNA levels of prePhHB and ACTIN in stems, leaves, and roots of transgenic and wild-type plants. ACTIN gene was used as an internal control. Transgenic lines (L1, L9, L10, L22, L25, L42 and L47) were transformed by Agrobacterium-mediated transformation containing the pLGNL-prePhHB. [file Image_4.jpeg]
