# Supplementary material for: The Biosynthesis of Heterophyllin B in Pseudostellaria heterophylla From prePhHB-Encoded Precursor
Source: Front Plant Sci. 2019 Oct 17;10:1259. doi: 10.3389/fpls.2019.01259 (PMC6842982; doi:10.3389/fpls.2019.01259)
Supplement: Supplementary file 5 [file Table_1.doc]

Table S1 Location of all collected samples

| Material number | Collection site | altitude/m | longitude /° | latitude /° |
| --- | --- | --- | --- | --- |
| 1 | Fujian Province,  China | 92 | 119.5521 | 27.2567 |
| 2 |  | 558 | 119.573 | 27.3816 |
| 4 |  | 84 | 119.7018 | 27.2248 |
| 5 |  | 635 | 119.7767 | 27.2468 |
| 6 |  | 853 | 119.8198 | 27.2754 |
| 7 |  | 683 | 119.9031 | 27.2032 |
| 8 |  | 1041 | 119.9288 | 27.1418 |
| 9 |  | 556 | 119.8598 | 27.1223 |
| 10 |  | 603 | 119.8689 | 27.0502 |
| 11 |  | 72 | 119.9722 | 27.2421 |
| 12 |  | 417 | 120.0046 | 27.1874 |
| 13 |  | 578 | 120.0356 | 27.2565 |
| 14 | Jiangsu Province,  China | 10 | 119.3121 | 32.1681 |
| 15 |  | 142 | 119.2863 | 31.7178 |
| 16 |  | 55 | 119.2683 | 31.6809 |
| 17 | Anhui Province,  China | 143 | 119.1887 | 31.3618 |
| 19 |  | 45 | 116.9111 | 31.3784 |
| 20 |  | 14 | 117.0169 | 31.4515 |
| 21 |  | 42 | 116.3838 | 31.7395 |
| 22 |  | 102 | 119.5363 | 30.8157 |
| 23 |  | 83 | 118.8 | 30.8052 |
| 24 |  | 50 | 118.7949 | 30.8637 |
| 26 |  | 50 | 119.2494 | 30.92 |
| 27 | Guizhou Province,  China | 776 | 118.0884 | 26.9878 |
| 28 |  | 1270 | 105.923 | 26.9885 |
| 29 |  | 520 | 109.1167 | 27.409 |
| 30 |  | 1076 | 108.022 | 27.2158 |
| 31 |  | 778 | 108.1773 | 27.0689 |
| 32 |  | 1252 | 106.2601 | 26.7667 |
| 33 |  | 860 | 107.8784 | 26.1817 |
| 34 |  | 780 | 107.2704 | 25.9278 |
| 35 |  | 934 | 107.9253 | 27.1402 |
| 36 |  | 1078 | 106.5864 | 26.2742 |
| 37 |  | 649 | 108.3457 | 27.0058 |
| 38 |  | 1016 | 107.464 | 26.7735 |
| 39 |  | 1100 | 108.24 | 27.0647 |
| 40 |  | 780 | 107.9029 | 27.2128 |
| 41 |  | 640 | 108.2214 | 27.0433 |
| 42 |  | 970 | 107.8918 | 27.1316 |
| 43 | Shandong Province, China | 56 | 118.5475 | 34.9143 |
| 44 |  | 66 | 118.3435 | 34.921 |
| 45 |  | 137 | 118.5727 | 35.3567 |
| 46 |  | 71 | 118.5049 | 34.9867 |
