# Supplementary material for: The Biosynthesis of Heterophyllin B in Pseudostellaria heterophylla From prePhHB-Encoded Precursor
Source: Front Plant Sci. 2019 Oct 17;10:1259. doi: 10.3389/fpls.2019.01259 (PMC6842982; doi:10.3389/fpls.2019.01259)
Supplement: Supplementary file 6 [file Table_2.doc]

Table S2 List of *P. heterophylla* samples

| No. | Source | Geographic location | Abbreviation |
| --- | --- | --- | --- |
| 1 | Jurong, Jiangsu,China | 119°17′10″N, 31°43′4″E | JSJR |
| 2 | Zherong, Fujian, China | 119°49′11″N, 27°16′31″E | FJZR |
| 3 | Xuanzhou, Anhui, China | 118°48′N, 30°48′18″E | AHXZ |
| 4 | Shibing, Guizhou, China | 108°1′19″N, 27°12′56″E | GZSB |
